# Development of Vaccines against Visceral Leishmaniasis

**DOI:** 10.1155/2012/892817

**Published:** 2011-09-05

**Authors:** Krystal J. Evans, Lukasz Kedzierski

**Affiliations:** The Walter and Eliza Hall Institute of Medical Research, The University of Melbourne, 1G Royal Parade, Parkville, VIC 3052, Australia

## Abstract

Leishmaniasis is a neglected disease resulting in a global morbidity of 2,090 thousand Disability-Adjusted Life Years and a mortality rate of approximately 60,000 per year. Among the three clinical forms of leishmaniasis (cutaneous, mucosal, and visceral), visceral leishmaniasis (VL) accounts for the majority of mortality, as if left untreated VL is almost always fatal. Caused by infection with *Leishmania donovani* or *L. infantum*, VL represents a serious public health problem in endemic regions and is rapidly emerging as an opportunistic infection in HIV patients. To date, no vaccine exists for VL or any other form of leishmaniasis. In endemic areas, the majority of those infected do not develop clinical symptoms and past infection leads to robust immunity against reinfection. Thus the development of vaccine for *Leishmania* is a realistic public health goal, and this paper summarizes advances in vaccination strategies against VL.

## 1. Introduction

There is currently no vaccine available for any form of leishmanias, including visceral leishmaniasis (VL), which if left untreated is almost always fatal. VL results from systemic infection with *L. infantum* (also known as *L. chagasi*) [1] which occurs in Europe, North Africa, South and Central America, and *L. donovani*, which is found throughout East Africa, India, and parts of the Middle East. Infection is initiated by the bite of an infected sand fly vector and parasites disseminate from the site of infection in the skin to reside and multiply within macrophages of the liver, spleen, and bone marrow [2]. VL, also known as kala-zar, is associated with fever, weight loss, enlargement of the spleen and liver, and anaemia. Leishmaniasis has strong links with poverty [3] and is considered one of the most neglected tropical diseases. Each year there are approximately 500,000 new cases of VL with over 90% of cases arising in India, Bangladesh, Nepal, Sudan, and Brazil. VL has become a frequent coinfection in HIV-positive individuals in endemic areas and is associated with enhanced onset of AIDS-related illness and increased VL treatment failure. Current VL therapy is based on the long-term parenteral administration of pentavalent antimonials, which, despite being expensive and highly toxic, has been the standard treatment for over 50 years.

Following *L. donovani* infection some individuals develop Post-kala-azar Dermal Leishmaniasis (PKDL), a complication that occurs either during or after treatment. During PKDL parasites reappear in the skin resulting in an array of small skin lesions, and patients are considered a significant infection reservoir because of the large number of parasites accessible to sand fly bites. Thus the treatment and control of PKDL are an important public health measure for controlling VL and must be considered in the development of VL vaccine strategies.

Host resistance to *Leishmania *infection is mediated by cellular immune responses leading to macrophage activation and parasite killing. Antileishmanial immunity is mediated by both innate and adaptive immune responses and requires effective activation of macrophages, dendritic cells (DCs), and antigen-specific CD4^+^ and CD8^+^T cells [4]. Although strong humoral responses are induced by VL infection, antibodies play no role in protection and are often associated with disease exacerbation [5]. Effector CD4^+^ T cells are responsible for the production of cytokines critical for the activation of macrophages and are required for optimal host response to infection [6]. Cytotoxic CD8^+^ T cells also play a host protective role, and are required for the effective clearance of parasites [7] and the generation of memory responses [8]. Interestingly, 80 to 90% of human infections are subclinical or asymptomatic, and this asymptomatic infection is associated with strong cell-mediated immunity. Only a small percentage of infected individuals develop severe disease, and patients who recover from VL display resistance to reinfection. This suggests the development of clinical immunity and provides a biological rationale for the development of VL vaccines that impart a strong cellular immunity.

Humans are the only known hosts for *L. donovani*; however *L. infantum* is primarily a zoonotic disease and canine species are the main animal reservoir. Canine visceral leishmaniaisis (CVL) affects millions of dogs in Europe, Asia, North Africa, and South America and has been associated with outbreaks of human VL [9]. Both symptomatic and asymptomatic *Leishmania*-infected dogs act as a source of parasites for VL transmission [10], and CVL represents a significant public health issue. A current approach to breaking the VL transmission cycle is the development of CVL vaccine, which may be crucial for controlling VL infection in human populations. 

Vaccination against VL has received limited attention compared with cutaneous leishmaniasis (CL). Historically CL has been the focus of vaccination attempts, as it has been known for centuries that people who resolve a primary CL skin lesion are protected from further infections. It is generally acknowledged that human VL trials will follow on from any successful CL immunization programme. Ideally a vaccine would provide cross-protection against multiple *Leishmania* species. The recent comparative genomic analysis of three *Leishmania* species, which cause distinct disease pathologies, showed that *L. major*, *L. braziliensis,* and *L. infantum* genomes are highly conserved and have very few species-specific genes [11]. The level of amino acid conservation within coding regions is also high between species, suggesting that the major *Leishmania* antigens are conserved and that a pan species vaccine may be achievable. However there is a high degree of variability in the cross-protective immunity induced by infection with different *Leishmania* species [12, 13] and VL-specific vaccines may provide a more successful intervention. 

Experimental infection models are used to screen and evaluate VL vaccines, and several animal species have been used including mice, hamsters, monkeys, and dogs [14]. However no single *in vivo* model accurately reflects all aspects of human VL disease, which has been a major limitation in the development of VL vaccines. The precise immune mechanisms underlying human VL are still not fully understood, and the responses necessary for protection by vaccination in experimental infection models may not reflect those required for efficacy in endemic areas [15].

The profile of an antileishmanial vaccine would need to incorporate several important features, such as safety, ease of production at a low cost in endemic countries, the induction of robust, long-term T cell responses, and both prophylactic and therapeutic efficacy. Ideally, such vaccine would offer cross-species effectiveness against CL and VL. As this might not be feasible, the development of a VL-specific vaccine remains an important global health priority.

## 2. First-Generation Vaccines

The only successful intervention against leishmaniasis is inoculation using virulent parasites, a process known as leishmanization (LZ). This ancient practise involves the administration of cutaneous *Leishmania* parasites to a discrete skin location, allowing a self-healing lesion to form. Initial immunological exposure then protects the individual from further infection and lesion development. LZ was traditionally practised by directly transferring infectious material from cutaneous lesions to uninfected individuals. However the establishment of an in vitro culture system in the early 20th century led to the large-scale production of promastigote forms of *Leishmania* for expanded clinical use. LZ induces a controlled, but full, infection and was successfully used as a prophylaxis throughout the Soviet Union, Asia, and the Middle East, with reported efficacy levels up to 100% [16, 17]. However LZ was largely abandoned due to safety issues associated with the use of live vaccines. Also, standardisation of the inoculum proved difficult as parasites used for LZ experience a dramatic loss of infectivity when subject to repeated subculturing [18]. Infection with live *Leishmania* also causes immunosuppression, which resulted in reduced immune responses to childhood vaccines and threatened the efficacy of immunization programmes [19, 20]. Currently only one country, Uzbekistan, employs the use of LZ, where a mixture of live and dead *L. major *is licensed as a vaccine for high-risk populations [21]. As LZ is the only vaccine strategy against *Leishmania* with proven efficacy in humans, efforts are being made to improve the safety of this practise. The inclusion of killed parasites in the inoculum and the use of adjuvants that promote rapid immune responses reduce the severity of primary lesions and accelerate wound healing during LZ [16, 22].

Research into first-generation vaccines based on whole-cell, killed *Leishmania* parasites dates back to the late 1930s, when pioneering work by Brazilian scientists demonstrated that killed parasites showed efficacy as both therapeutic and prophylactic vaccines [23]. Over the ensuing decades numerous preparations of killed parasites were tested, either alone or in combination with a variety of different adjuvants. Although displaying well-tolerated safety profiles, to date no first-generation vaccine using killed parasites has demonstrated sufficient efficacy as a prophylactic vaccine to be used in widespread control programmes [24]. Most vaccine studies focus on CL, and there have been no clinical trials of first-generation vaccines produced from VL *Leishmania* species. Due to the strongly conserved genomes of the *Leishmania *species, it is anticipated that human VL trials will follow any successful CL immunization program. Whether the same vaccine can show efficacy against both CL and VL remains to be determined. Interestingly, killed parasite vaccines using an alum-precipitated autoclaved *L. major *(ALM) given with a BCG adjuvant have shown promise as vaccines for VL and PKDL [25]. When given to patients with persistent PKDL in combination with antimonial therapy this vaccine showed enhanced cure rates and lower incidence of relapse as compared to antimonial treatment alone [26]. Based on these initial studies, recommendations have been put forward for expanded trials to examine the prophylactic and therapeutic effects of the alum-ALM + BCG vaccine for PKDL and VL [27].

Evidence from experimental animal models supports the development of first-generation VL vaccines. Complete soluble antigen (CSA) from an attenuated *L. donovani* strain was effective as both a therapeutic and prophylactic vaccine in susceptible mice, without the use of an adjuvant [28]. Importantly, CSA immunization was effective against both pentavalent antimony sensitive and resistant strains of *L. donovani. *Vaccination with purified excreted-secreted antigens from *L. infantum* promastigotes (LiESAp) fully protected dogs from experimental challenge and induced a long-lasting cell-mediated immunity [29].

A major advantage of first-generation vaccines is that they are conceptually simple and relatively easy to produce in *Leishmania* endemic countries at low cost. However standardization of vaccines derived from cultured parasites is difficult, and this has hindered commercial development efforts. The route of administration, formulation, and adjuvant are also important considerations in the development of whole-parasite vaccines, and optimisation is essential for the induction of protective immune responses. The most recent clinical trials of first-generation vaccines have demonstrated a good safety profile but have not conferred significant levels of protection for use as prophylactic vaccines. However promising results from trials using therapeutic vaccination in combination with chemotherapy warrant further investigation.

## 3. Second-Generation Vaccines

The development of Second-generation vaccines for *Leishmania* has included recombinant proteins, polyproteins, DNA vaccines, liposomal formulation, and dendritic cell vaccine delivery systems. A variety of different molecules have been tested to date with varying degrees of efficacy (Table 1). In general VL vaccination studies have been hampered by the lack of a suitable animal model. The natural combination of dogs and *L. infantum *[30] and *L. donovani *in golden hamsters [31] reproduces many features of human VL. The canine model is particularly useful in evaluating vaccine candidates since successful vaccination of dogs might control the spread of disease to humans in endemic areas where the dog is the reservoir of the parasite [32]. However, both models suffer from lack of immunological reagents and assays needed for the characterisation of immune responses. Therefore, the mouse model of VL has been widely used to assess vaccine candidates. While experimental VL infection in mice does not fully reproduce the disease observed in humans, mice are competent hosts for both *L. donovani* and *L. infantum* and exhibit organ-specific pathology in the liver and spleen. Other major advantages of the mouse model are that it is amenable to genetic manipulation to create mutants with specific deficiencies in the immune system and a wide range of immunological reagents is available.

Only a small number of recombinant proteins have been tested against VL in murine models. Early studies showed that promastigote-derived membrane protein dp72 protected mice against *L. donovani *infection [59, 82], but there has been no further advance on the use of this antigen for the development of vaccines. The *L. donovani *amastigote LCR1 protein containing 67-amino-acid repeats homologous to repeats in a *Trypanosoma cruzi* flagellar polypeptide was tested for protection in mice. Recombinant protein led to partial protection against *L. infantum *challenge [44], while immunization with BCG-LCR1 elicited better protection [45]. Vaccine efficacy was influenced by the site of immunization with subcutaneous administration superior to intraperitoneal inoculation [45]. Recombinant hydrophilic acylated surface protein B1 (HASPB1), a member of a family of proteins expressed only in metacyclic and amastigote stages, has shown efficacy in an experimental mouse model of VL [8]. This vaccine did not require the use of adjuvant, and protection was associated with the induction of antigen-specific, IFN-*γ* producing CD8^+^ T cells, a mechanism similar to DNA vaccination [8]. Immunization with the *L. donovani* A2 cysteine proteinase delivered as recombinant protein or as DNA also afforded protection against experimental challenge infection [60, 61]. Other antigens tested include amastigote cysteine proteases (CPs) [40], kinetoplastid membrane protein-11 (KMP-11) [65], amastigote LCR1 [45], leishmanial antigen ORFF [63], and NH36, a main component of the fucose-mannose ligand [47]. 

Apart from defined single molecules, multicomponent vaccines have been shown to protect against VL in experimental infection systems. Recombinant Q protein formed by fusion of antigenic determinants from four cytoplasmic proteins from *L. infantum *(Lip2a, Lip2b, P0, and histone H2A) coadministered with live BCG protected 90% of immunised dogs by enhancing parasite clearance [49]. To date, only one multicomponent vaccine, Leish-111f, has been assessed in clinical trials [83]. Leish-111f is a single polyprotein composed of three molecules fused in tandem: the *L. major *homologue of eukaryotic thiol-specific antioxidant (TSA), the *L. major* stress-inducible protein-1 (LmSTI1), and the *L. braziliensis* elongation and initiation factor (LeIF) [83]. There is some evidence that the Leish-111f vaccine can also induce partial protection against VL in animal models [56]; however, it failed to protect dogs against infection and did not prevent disease development in a Phase III vaccine trial in dogs [55]. An optimized version, known as Leish-110f, has recently demonstrated strong immunogenicity and some protective efficacy against *L. infantum* in mice [84]. The Leish-111f vaccine is moving forward into clinical trials as LeishF1 and is being trialled in combination with the MPL-SE adjuvant. This adjuvant consists of monophosphoryl lipid A, a potent TLR4 agonist, formulated with the antigen as a stable emulsion. A recent small-scale clinical trial in a *L. donovani* endemic area showed Leish-F1-MPL-SE was safe and well tolerated in people with and without prior VL exposure and induced strong antigen-specific T cell responses [85]. 

In addition to recombinant proteins, DNA has been extensively tested as means of vaccine delivery. The induction of Th1 responses leading to strong cytotoxic T cell immunity is a general property of DNA vaccines [86], and a growing body of evidence implicates CD8^+^ T cells in protective antileishmanial responses [87]. The PapLe22 antigen, which is recognised by T cells from VL patients [88], was administered as a DNA vaccine and led to a marked decrease in parasite burden in immunised hamsters [46]. However stimulation of peripheral blood mononuclear cells from VL-infected individuals with recombinant PapLe22 induced IL-10 production [88], which is associated with VL pathogenesis in humans [89]. The *Leishmania* homologue for receptors of activated C kinase (LACK) is the most extensively studied DNA vaccine against both cutaneous and visceral leishmaniasis, but has shown inconsistent outcomes. DNA vaccination with a plasmid harbouring the LACK gene coadministered with, or without, IL-12 induced robust, long-lasting protection against *L. major *challenge in mice, which was dependent on CD8^+^ T cells [90–92]. In a heterologous system, priming with *L. infantum* LACK followed by a vaccinia booster afforded protection against *L. major *infection [93]. The prime-boost regimen was also employed to immunise dogs against *L. infantum* infection and elicited protective responses in 60% of vaccinated animals [38], but this positive outcome has been overshadowed by studies where immunisation with LACK offered no protection. In an experimental mouse model the LACK DNA vaccine induced strong Th1 responses, but failed to protect against *L. donovani* challenge [58]. Other studies in the *L. infantum *mouse model confirmed that LACK DNA vaccination does not confer protection against VL despite the presence of Th1 responses [36]; however, a strategy using a heterologous prime-boost vaccination using DNA and vaccinia viruses has shown some efficacy [35]. The heterologous DNA-prime protein-boost approach has also shown success for other VL vaccine antigens such as ORFF [63] and cysteine proteinases [94]. Heterologous prime-boost with gp63 antigen with CpG-ODN as adjuvant provided durable protection against *L. donovani* challenge in an experimental mouse model and was associated with robust cellular immune responses [95]. As gp63 is a major surface protein present on both amastigote and promastigote forms and shows a high homology between VL species [96] it is an attractive target for further vaccine development.

Insights into the role of the innate immune system, in particular dendritic cells (DCs), have provided the impetus for the use of DCs as a delivery system for *Leishmania* antigens [97–99]. DCs loaded with *L. donovani *soluble extract and expressing high levels of IL-12 induced protection in the mouse model of VL when used as a therapeutic vaccine [100]. Moreover, coadministration of DCs with antimonial therapy resulted in complete clearance of parasites from the liver and spleen, unlike DC immunisation alone which was not able to clear the infection from these organs [101]. 

Liposome formulations have been adopted as *Leishmania* drug delivery systems, and liposomal Amphotericin B is the current preferred drug treatment for VL in resource-rich settings [102]. Vesicle delivery systems are also being considered for VL vaccines and have been shown to adjuvant protein antigens and induce sustained Th1 immune responses [103]. These delivery systems have shown some protection against *L. donovani* infection in experimental mouse models [104] and provide a new approach to the development of VL vaccines. 

Recently, Peters et al. [105] demonstrated that sand fly transmission of parasites abrogates vaccine-induced protective immunity. While mice vaccinated with killed parasites were refractory to a needle challenge, they were susceptible to the sand fly inoculum implying that the protective responses in vaccinated mice were either not generated or not maintained. These data provide a rationale for the inclusion of sand fly saliva components, which are specific to natural infection, in vaccine design. The sand fly injects Leishmania parasites in the presence of saliva, which contains a range of pharmacologically active molecules that can modulate host's immune and inflammatory responses and facilitate establishment of infection. For a number of years salivary gland antigens have been targeted as potential candidates for antileishmanial vaccine development, primarily against *L. major*. Nevertheless, it has been shown that children who underwent anti-*VL *delayed-type hypersensitivity (DTH) conversion also had increased titers of antibodies directed to sand fly saliva suggesting that mounting an effective antileishmanial response might be linked to neutralization of saliva components [106].

## 4. Development of Canine Vaccines

Eliminating animal reservoirs has been an essential public health tool for the control of many zoonotic diseases, such as rabies [107] and brucellosis [108]. Canines, particularly domestic dogs, are the main reservoir for VL species and are considered the main source of zoonotic transmission to humans. The development of an effective canine visceral leishmaniasis (CVLs) vaccine represents a cost-effective tool for interrupting the transmission cycle and controlling zoonotic VL infection in humans.

CVL is widespread throughout South America [9] and the Mediterranean [109] where *L.infantum* is the most significant causative agent of disease. *L. donovani* is considered to be zoonotic, but as yet there has been no clear identification of the reservoir host animal [110]. Asymptomatic infection is common in dogs, and as a large reservoir of parasites are present in the skin, asymptomatic animals are a major source of infection for vector transmission [10]. Human VL is an emerging disease in many areas of the world, including Northern Europe [111] and North America [112], and the spread of VL into nonendemic areas is often preceded by increased incidence of canine infection. There is concern that increased mobility of dogs and changes in vector habitat will result in increased transmission of human VL in previously nonendemic areas [113].

Treatment of CVL shows low efficacy with drugs successfully used for human VL chemotherapy, and drug treatment of dogs rarely results in cure [114]. Control programmes for CVL have a demonstrated capacity to reduce the prevalence of human VL disease following interventions that target dog populations in endemic regions [115]. However these public health campaigns are often complex and expensive to maintain, leading to varying degrees of efficacy. The use of insecticide-impregnated collars can reduce the risk of contracting CVL [116], but is costly and difficult to implement at a national level. The culling of seropositive dogs has long been recommended in Brazil; however this approach has not lead to a reduction in the number of human VL cases and may be of limited value [117]. Therefore the development of vaccines against CVL is an attractive approach to controlling infection in dogs, reducing the parasite reservoir and thus reducing the risk of transmission of VL to human populations.

Immunological characterisation of CVL reveals cellular and humoral immune responses comparable to human infection, including immune dysregulation and increased IL-10 which is associated with disease manifestation and progression [118]. Disease resistance is associated with strong Th1-type immune responses, including IFN-*γ* expression by antigen-specific T cells. Thus, analogous to a human VL vaccine, an effective CVL vaccine needs to induce strong and long-lasting cell-mediated immunity. Adjuvant choice must be carefully considered for CVL interventions, as live BCG is not appropriate for use in dogs and the identification of appropriate and effective adjuvants will be essential for safe and effective CVL vaccines [119]. In addition, sand fly components are being considered for inclusion in CVL vaccine. Reactive antibodies to two sand fly saliva components (LuLo-D7 and LuLo YELLOW) were identified in infected dogs and proposed as possible vaccine candidates against CVL [120]. Evaluation of a killed *Leishmania *vaccine containing sand fly saliva extract indicated that the vaccine is highly immunogenic and provided support for further development of saliva components as candidates for anti-VL vaccine [121]. This is supported by vaccination studies using the hamster VL model, showing that salivary protein LJM19 was able to protect hamsters from fatal infection with *L. infantum * [122]. In addition, immunization with salivary proteins LJM17 and LJL143 induced strong cellular and humoral responses in dogs and might be an advantageous addition to anti-CVL vaccine [123].

Currently there are two commercially available CVL vaccines, Leishmune and Leishtec, and new vaccines under development include recombinant antigen vaccines and both live and killed whole-cell vaccines. 

The Leishmune vaccine was the first commercially licensed vaccine for CVL, produced by Fort Dodge Animal Health and has been available in Brazil since 2004 [124]. This vaccine consists of the fucose mannose ligand (FML) isolated from *L. donovani* plus a saponin adjuvant. FML is a glycoprotein mixture, and the surface glycoconjugate GP36 is the major immunogen component [125]. This vaccine induced a significant and strong protective effect during phase III trials in dogs living in a VL-endemic area in Brazil with a vaccine efficacy as high as 80% [77, 126]. This protection lasted up to 3.5 years following vaccination, indicating induction of a long-lasting immunity [77]. As Leishmune-vaccinated dogs showed a complete absence of parasites, this renders them noninfectious and contributes to the breakdown of the zoonotic VL transmission cycle [79]. During phase III trials of Leishmune there was a concomitant reduction in human VL cases in districts where dogs were vaccinated [126] demonstrating that CVL vaccination interrupts the transmission of disease to humans. FML antigens are present on the surface of *Leishmania *parasites throughout the life cycle, and antibodies raised in vaccinated dogs prevented the binding of procyclic promastigotes to the sand fly midgut [78]. Thus Leishmune acts as a transmission blocking vaccine by clearing parasites from the animal reservoir and preventing survival of the parasite in the sand fly vector. Currently the Leishmune vaccine is used as a prophylactic and is recommended for asymptomatic noninfected dogs. However studies show that Leishmune is effective as a therapeutic vaccine for naturally infected dogs [127], particularly when given in combination with chemotherapy [128]. Emerging wide-scale field studies reveal that Leishmune decreases the incidence of both human and canine visceral leishmaniasis after dog vaccination with Leishmune [129].

A second vaccine, known as Leish-Tec, is being commercially developed by the Hertape Calier Saúde Animal and consists of adenovirus expressing the *L. donovani *A2 antigen. Whilst the results from phase-III trials of Leish-Tec are yet to be published it is known that immunization with a recombinant A2 protein elicits protection against the onset of clinical VL in experimental dog infections [51]. The recombinant adenovirus encoding the *A2 *gene was capable of inducing strong Th1-type immune responses in vaccinated mice and reduced parasite burdens following challenge with VL parasites [130]. Together these studies indicate that A2 is an important candidate antigen for the development of CVL vaccines, and future studies should report on the impact of this intervention on both canine and human VL infection.

As many of the clinical and immunological features of CVL are similar to those observed in human VL, experimental challenge in dogs represents a useful system for evaluating the efficacy of vaccine candidates. The Leish-111f +  MPL-SE vaccine is a leading vaccine candidate from human VL and has shown therapeutic efficacy in recent CVL trials [57]. Live attenuated parasites vaccines are also being explored in canine models, including a drug-attenuated line of *L. infantum *established by culturing promastigotes under gentamicin pressure. The attenuated *L. infantum *vaccine strain did not induce clinical symptoms of VL in dogs and provided protection from subsequent challenge with live virulent *L. infantum * [131]. 

The elimination of human VL will be difficult to achieve in the presence of persisting animal reservoirs, and veterinary intervention is an important tool for reducing the global burden of human VL disease. The identification of measurable and reliable biomarkers of immunogenicity and protection induced by CVL vaccines may also be informative for human VL vaccine efforts.

## 5. Live Attenuated Vaccines

Historically the most successful vaccines against intracellular pathogens have been based on live attenuated organisms. Vaccination strategies using live attenuated *Leishmania *parasites are attractive as they closely mimic the natural course of infection and may elicit clinically protective immune responses. A live attenuated vaccine strain would present a full complement of *Leishmania *antigens to the host immune system along with appropriate pattern-recognition molecules for the parasite. Live vaccines also deliver antigens to the correct cellular and tissue compartments for appropriate processing and presentation to the host immune system. Together, this enhances the capacity of live attenuated vaccines to promote antigen-specific effector and memory immune responses that confer long-lasting protective immunity.

The development of robust *in vitro *culture systems for growth and differentiation of *Leishmania* promastigote and amastigote life cycle stages has enabled the production of attenuated vaccine strains. It should be noted that most research in this area has utilized CL strains, such as *L. major*; however the attenuation techniques are broadly transferrable to VL causing species. It is has been known for some time that long-term *in vitro* culture of promastigote parasites leads to a loss of virulence *in vivo*. Studies in experimental mouse models of CL have shown that infection with cloned avirulent lines provides clear protection against a virulent challenge infection [132]. Avirulent strains of the VL species *L. donovani* and *L. infantum* have been generated by repeated *in vitro* subculture of promastigotes in the presence of gentamicin [133]. These drug-attenuated promastigotes were able to invade macrophages but could not survive as intracellular amastigote forms [133]. Drug-attenuated *L. infantum *was avirulent in an experimental canine model, induced strong cellular immunity production and protection against challenge with live virulent *L. infantum * [131]. Early experiments showed that *γ*-irradiation rendered *Leishmania* parasites nonpathogenic and infection protected against challenge in a cutaneous *Leishmania* model. Protection depended on the presence of viable irradiated parasites, suggesting that transformation into amastigote forms is required for efficacy. Interestingly the underlying mechanism of protection may relate more to the induction of tolerization rather than immunization in this system [134]. Other approaches to the generation of attenuated parasites include chemical mutagenesis screens selecting for temperature sensitive CL strains [135] which are avirulent during infection and significantly protect against subsequent challenge.

The major concern regarding these approaches to attenuation is that the underlying genetic mechanisms are not defined. This creates safety concerns as the stability of parasite attenuation is uncertain and parasites could revert to a virulent form. Conversely, a progressive loss of virulence may occur, resulting in parasite lines that are incapable of establishing infection or inducing protective host responses. A loss of parasite virulence due to long-term *in vitro* culture has been demonstrated in both human patients undergoing leishmanization and experimental mouse models [19]. Thus in the absence of a clear genetic profile, nonspecific parasite attenuation is not acceptable for the development of a human VL vaccine.

Over the last few decades the development of a powerful “genetic toolkit” for *Leishmania* species has enabled research involving transgenic parasites [136]. *L. donovani* and *L. infantum* parasites can be stably transfected using integrating expression constructs that target genes for disruption by homologous recombination. As *Leishmania* organisms are diploid throughout their lifecycle, the production of null mutants requires each allele of a gene to be targeted individually with genetic constructs containing two different and independent selectable markers. The recent availability of *Leishmania *genome sequences has facilitated the identification and in-depth analysis of parasite genes crucial for infection and virulence. Comparative genomics studies of *Leishmania *species have shown a highly similar gene content and gene order and annotation studies have revealed only a few species-specific genes [11]. Increased knowledge of potential parasite virulence factors and a greater understanding of the antigens involved in the acquisition of immunity have generated much interest in the development of genetically attenuated parasite vaccines.

To date, there have been two general approaches to the genetic attenuation of *Leishmania *parasites. First, by deletion of genes encoding virulence factors or the enzymes responsible for their synthesis and second, by targeting genes essential for intracellular survival. Gene targeting aims to produce parasites that are capable of being produced and manipulated *in vitro*, usually in promastigote form, but incapable of sustaining virulent infection in the host, in amastigote form. The first genetically attenuated parasite vaccine was the *L. major* dihydrofolate reductase-thymidylate synthase (dhfr-ts) knockout, which targeted an essential metabolic gene [137]. This null mutant was able to establish a persistent infection in experimental mouse models, but remained avirulent with respect to disease. Importantly vaccination with dhfr-ts knockout parasites elicited substantial protective immunity, as mice were resistant to subsequent challenge with virulent *L. major*. Although further experiments in nonhuman primate models failed to show protection these initial studies provided proof of principle for the safety and immunogenicity of live attenuated *Leishmania* vaccines [138]. Drug-sensitive *Leishmania* mutants containing suicide genes [139–141] are also being developed for use during leishmanization, and inducible suicide mutants in *L. amazonensis* have shown protective efficacy in an experimental hamster model [142].

To date, only a small number of studies have focused on generating attenuated forms of the VL species *L. infantum* and *L. donovani *as a route to the production of an attenuated VL vaccine. One approach has targeted the transporters for the metabolic precursors of the folate pathway, as *Leishmania* parasites are auxotrophic for folate and pterin [143]. *L. donovani *parasites lacking the main biopterin transporter (BT1) showed a marked reduction in infectivity in an experimental mouse model, and this attenuated strain conferred protection to subsequent challenge with wild-type *L. donovani * [144]. Parasites incapable of intracellular reproduction were produced by targeting centrin, a calcium binding cytoskeletal protein. A loss of centrin from *L. donovani* parasites did not affect the growth of promastigote forms, but null mutants were unable to survive as axenic amastigotes or in human macrophages *in vitro *[145]. Immunization of mice and hamsters by infection with centrin deficient *L. donovani* protected against virulent homologous challenge. Importantly the centrin null vaccine strain elicited parasite-specific Th1 responses which strongly correlated with sustained protection and also induced a level of cross protection against *L. braziliensis* infection [146].

The genetic attenuation of *Leishmania* does not necessarily require the production of null mutants. Deletion of one allele of the *L. infantum* silent information regulatory 2 (SIR2) locus was sufficient to prevent amastigotes from undergoing intracellular replication in macrophages. Immunization with *L. infantum* lacking one SIR2 gene copy elicited strong parasite-specific T cell responses and conferred complete protection against virulent challenge in a VL mouse model [147].

Other approaches to developing live attenuated parasites as VL vaccines have utilised nonpathogenic *Leishmania* species, an approach comparable to the use of BCG as a vaccine against *Mycobacterium tuberculosis* infection. The lizard protozoan parasite *L. tarentolae* has never been found to be associated with any human leishmaniasis and is considered nonpathogenic. Whilst *L. tarentolae* is capable of infecting mammalian cells and transforming into amastigotes, the parasite does not cause clinical symptoms of disease in either mouse or hamster models [148]. In experimental vaccine trials *L. tarentolae* elicited a strong Th1-driven protective immune response and conferred protection against infectious challenge with *L. donovani* in a susceptible mouse strain [149]. The use of *L. tarentolae* as a vaccine vector to deliver specific *Leishmania* antigens in the context of a live infection has also been explored. The *L. donovani* A2 antigen was expressed in *L. tarentolae*, which normally lacks this protein [150] and used as a vaccine strain in an experimental mouse model. Vaccination protected susceptible mice against *L. infantum* challenge and was associated with the production of high levels of IFN-*γ* production [151].

The use of live attenuated vaccines provides a promising vaccination strategy for VL; however safety issues regarding the use of genetically attenuated parasites as vaccines still need to be addressed. Many of the proposed live attenuated vaccines induce long-lasting immunity to reinfection by maintaining a low level asymptomatic infection. The establishment of subclinical infection is particularly valuable as the persistence of antigen is thought essential for the generation of effective memory responses to *Leishmania*. However reactivation of *Leishmania* has been observed in patients who are immunocompromised, such as following HIV infection, thus the safety of attenuated parasites that induce a subclinical infection will need to be carefully assessed.

Transgenic parasites provide an enticing lead for vaccine development. A continuing synergy between molecular and immunological approaches to the development of VL vaccines will accelerate development of the next generation of therapeutics. In addition, transgenic parasites are invaluable tools for understanding host-parasite interactions [152] and inform vaccine design by providing insight into immunity and pathogenesis during VL.

## 6. Concluding Remarks

Preventive vaccines are recognized as the best and most cost-effective protection measure against pathogens and save millions of lives across the globe each year. *Leishmania *vaccine development has proven to be a difficult and challenging task and is hampered by an inadequate knowledge of disease pathogenesis, the complexity of immune responses needed for protection, and the cost of vaccine development. The burden of VL is concentrated in resource poor nations, and a lack of political will and philanthropic investment further aggravates the situation. However, the rise of biotechnology industries in endemic countries, such as India, may provide an impetus for VL vaccine development and investment. A recent clinical trial in India assessed the safety and immunogenicity of the LEISH-F1+MPL-SE vaccine [85] which is the only Second-generation vaccine currently in clinical development for human VL. There are currently several new European-based VL vaccine efforts including a synthetic vaccine RAPSODI (http://www.fp7-rapsodi.eu/) [153], a DNA-based LEISHDNAVAX (http://www.leishdnavax.org/) [154], and an adenovirus vectored therapeutic vaccine (Paul Kaye, personal communication). New adjuvants are also being developed, and there are several clinical vaccine trials in progress and in planning [18]. Given the rapid progress in the fields of parasite immunology and genomics, a successful anti-*Leishmania* vaccine should be achievable sooner rather than later. There is a clear need for greater investment in research and development to move promising vaccine leads along the development pathway toward an effective, affordable VL vaccine.

## Figures and Tables

**Table 1a:** (a)

Vaccines tested against *L. infantum *					
Antigen	Source of antigen	Vaccine delivery	Animal model	Outcome	Reference
KMPII, TRYP, LACK, and GP63	*L. infantum*	DNA vaccine	Dog	No protection	Rodríguez-Cortés et al., 2007 [33]
H2A, H2B, H3, and H4	*L. infantum*	DNA vaccine	Mouse	No protection	Carrión et al., 2008 [34]
p36 LACK	*L. infantum*	DNA vaccine + protein expressed in vaccinia virus	Mouse	Protection	Dondji et al., 2005 [35]
p36 LACK	*L. infantum*	DNA vaccine	Mouse	No protection	Marques-da-Silva et al., 2005 [36]
p36 LACK	*L. infantum*	DNA vaccine	Mouse	Protection	Gomes et al., 2007 [37]
p36 LACK	*L. infantum*	DNA vaccine + protein expressed in vaccinia virus	Dog	Protection	Ramiro et al., 2003 [38]
p36 LACK	*L. infantum*	DNA vaccine + protein expressed in vaccinia virus	Dog	Protection	Ramos et al., 2008 [39]
CPA and CPB	*L. infantum*	DNA vaccine + recombinant protein	Mouse	Protection	Rafati et al., 2006 [40]
CPA and CPB	*L. infantum*	DNA vaccine + recombinant protein	Dog	Protection	Rafati et al., 2005 [41]
CTE of CPIII	*L. infantum*	DNA vaccine + recombinant protein	Mouse	No protection	Rafati et al., 2008 [42]
CPC	*L. infantum*	DNA vaccine + recombinant protein	Mouse	Protection	Khoshgoo et al., 2008 [43]
LCR1	*L. infantum*	Recombinant protein	Mouse	Partial protection	Wilson, et al., 1995 [44]
LCR1	*L. infantum*	Protein expressed in BCG	Mouse	Partial protection	Streit et al., 2000 [45]
PapLe22	*L. infantum*	DNA vaccine	Hamster	Partial protection	Fragaki et al., 2001 [46]
NH36	*L. donovani*	DNA vaccine	Mouse	Partial protection	Aguilar-Be et al., 2005 [47]
FML	*L. donovani*	Protein	Mouse	Partial protection	Aguilar-Be et al., 2005 [47]
FML	*L. donovani*	Protein	Mouse	Protection	Oliveira-Freitas et al., 2006 [48]
Q protein	*L. infantum*	Recombinant fusion protein of Lip2a, Lip2b, P0, and H2A + BCG	Dog	Protection	Molano et al., 2003 [49]
Q protein	*L. infantum*	Recombinant fusion protein of Lip2a, Lip2b, P0, and H2A + BCG	Mouse	Partial protection	Parody et al., 2004 [50]
A2	*L. donovani*	Recombinant protein	Dog	Partial protection	Fernandes et al., 2008 [51]
A2 and NH	*L. donovani*	DNA vaccine	Mouse	Partial protection	Zanin et al., 2007 [52]
LiESAp	*L. infantum*	Native protein	Dog	Protection	Lemesre et al., 2005 [53] and 2007 [54]
LiESAp	*L. infantum*	Native protein	Dog	Protection	Bourdoiseau et al., 2009 [29]
Leish-111f	*L. major*	Recombinant polyprotein of TSA, LmSTI1, and LeIF	Dog	No protection	Gradoni et al., 2005 [55]
Leish-111f	*L. major*	Recombinant polyprotein of TSA, LmSTI1, and LeIF formulated in MPL-SE	Dog	Protection	Coler et al., 2007 [56]
Leish-111f	*L. major*	Recombinant polyprotein of TSA, LmSTI1, and LeIF formulated in MPL-SE	Dog	Protection	Trigo et al., 2010 [57]

CP: cysteine proteinase; CTE: C-terminal extension; BCG: *Mycobacterium bovis* bacillus Calmette-Guerin; SLA: soluble leishmanial antigens; FML: fucose-mannose ligand; LiESAp: Leishmania infantum excreted-secreted antigen purified; LPG: lipophosphoglycan.

**Table 1b:** (b)

Vaccines tested against *L. donovani *					
Antigen	Source of antigen	Vaccine delivery	Animal model	Outcome	Reference

p36 LACK	Multiple species	DNA vaccine	Mouse	No protection	Melby et al., 2001 [58]
dp72	*L. donovani*	Native protein antigen	Mouse	Partial protection	Jaffe et al., 1990 [59]
A2	*L. donovani*	Recombinant protein	Mouse	Protection	Ghosh et al., 2001 [60]
A2	*L. donovani*	DNA vaccine	Mouse	Protection	Ghosh et al., 2001 [61]
HASPB1	*L. donovani*	Recombinant protein	Mouse	Protection	Stager et al., 2000 [8]
ORFF	*L. donovani*	Recombinant protein	Mouse	Partial protection	Tewary et al., 2004 [62]
ORFF	*L. donovani*	DNA vaccine + recombinant protein	Mouse	Partial protection	Tewary et al., 2005 [63]
ORFF	*L. donovani*	DNA vaccine	Mouse	Partial protection	Sukumaran et al., 2003 [64]
KMP-11	*L. donovani*	DNA vaccine	Hamster	Protection	Basu et al., 2005 [65]
KMP-11	*L. donovani*	DNA vaccine	Mouse	Partial protection	Bhaumik et al., 2009 [66]
gp36	*L. major*	Recombinant protein expressed in bacilli	Mouse	Partial protection	McSorely et al., 1997 [67]
gp36	*L. donovani*	Native protein in cationic liposomes	Mouse	Partial protection	Bhowmick et al., 2008 [68]
SLA	*L. donovani*	Native proteins in cationic liposomes	Mouse	Protection	Bhowmick et al., 2007 [69]
SLA	*L. donovani*	Native proteins	Mouse	Protection	Tewary et al., 2004 [70]
LD9, LD72, LD51, LD31	*L. donovani*	Native proteins in cationic liposomes	Mouse	Protection	Bhowmick and Ali, 2009 [71]
Leishmanial antigens	*L. donovani*	Native proteins in liposomes	Mouse	Protection	Mazumdar et al., 2004 [72]
F14	*L. donovani*	Recombinant protein	Hamster	Partial protection	Bhardwaj et al., 2009 [73]
*γ*-GCS	*L. donovani*	DNA vaccine	Mouse	Partial protection	Carter et al., 2007 [74]
FML	*L. donovani*	Native protein	Mouse	Partial protection	Palanik-de-Sousa et al., 1994 [75]
FML	*L. donovani*	Native protein	Mouse	Partial protection	Santos et al., 1999 [76]
FML	*L. donovani*	Formulation with QuilA saponin	Dog	Protection	Borja-Cabrera et al., 2002 [77]
Leishmune (FML)	*L. donovani*	Recombinant protein	Dog	Partial protection	Saraiva et al., 2006 [78]
Leishmune (FML)	*L. donovani*	Recombinant protein	Dog	Protection	Nogueira et al., 2005 [79]
H2A, H2B, H3, H4 and LACK	*L. donovani*	Multiunit DNA vaccine	Dog	Partial protection	Saldarriaga et al., 2006 [80]
LPG	*L. donovani*	Purified glycolipid + BCG	Hamster and mouse	No protection	Tonui et al., 2003 [81]

CP: cysteine proteinase; CTE: C-terminal extension; BCG: *Mycobacterium bovis* bacillus Calmette-Guerin; SLA: soluble leishmanial antigens; FML: fucose-mannose ligand; LiESAp: Leishmania infantum excreted-secreted antigen purified; LPG: lipophosphoglycan.

## References

[1] Mauricio IL, Stothard JR, Miles MA (2000). The strange case of *Leishmania chagasi*. *Parasitology Today*.

[2] Leclercq V, Lebastard M, Belkaid Y, Louis J, Milon G (1996). The outcome of the parasitic process initiated by *Leishmania infantum* in laboratory mice: a tissue-dependent pattern controlled by the Lsh and MHC loci. *The Journal of Immunology*.

[3] Alvar J, Yactayo S, Bern C (2006). Leishmaniasis and poverty. *Trends in Parasitology*.

[4] Stanley AC, Engwerda CR (2007). Balancing immunity and pathology in visceral leishmaniasis. *Immunology and Cell Biology*.

[5] Kaye PM, Svensson M, Ato M (2004). The immunopathology of experimental visceral leishmaniasis. *Immunological Reviews*.

[6] Kharazmi A, Kemp K, Ismail A (1999). T-cell response in human leishmaniasis. *Immunology Letters*.

[7] Stern JJ, Oca MJ, Rubin BY, Anderson SL, Murray HW (1988). Role of L3T4+ and Lyt-2+ cells in experimental visceral leishmaniasis. *The Journal of Immunology*.

[8] Stager S, Smith DF, Kaye PM (2000). Immunization with a recombinant stage-regulated surface protein from *Leishmania donovani* induces protection against visceral leishmaniasis. *The Journal of Immunology*.

[9] Dantas-Torres F (2009). Canine leishmaniosis in South America. *Parasites and Vectors*.

[10] Lima LVR, Carneiro LA, Campos MB (2010). Canine visceral leishmaniasis due to *Leishmania* (L.) infantum chagasi in Amazonian Brazil: comparison of the parasite density from the skin, lymph node and visceral tissues between symptomatic and asymptomatic, seropositive dogs. *Revista do Instituto de Medicina Tropical de Sao Paulo*.

[11] Peacock CS, Seeger K, Harris D (2007). Comparative genomic analysis of three *Leishmania* species that cause diverse human disease. *Nature Genetics*.

[12] Lainson R, Shaw JJ (1977). Leishmaniasis in Brazil:XII. Observations on cross immunity in monkeys and man infected with *Leishmania mexicana* mexicana, L. m. amazonensis, L. braziliensis braziliensis, L. b. guyanensis and L. b. panamensis. *Journal of Tropical Medicine and Hygiene*.

[13] Porrozzi R, Teva A, Amaral VF, Santos da Costa MV, Grimaldi G (2004). Cross-immunity experiments between different species or strains of *Leishmania* in rhesus macaques (Macaca mulatta). *American Journal of Tropical Medicine and Hygiene*.

[14] Garg R, Dube A (2006). Animal models for vaccine studies for visceral leishmaniasis. *Indian Journal of Medical Research*.

[15] Nylen S, Gautam S (2010). Immunological perspectives of leishmaniasis. *Journal of Global Infectious Diseases*.

[16] Khamesipour A, Dowlati Y, Asilian A (2005). Leishmanization: use of an old method for evaluation of candidate vaccines against leishmaniasis. *Vaccine*.

[17] Nadim A, Javadian E, Tahvildar-Bidruni G, Ghorbani M (1983). Effectiveness of leishmanization in the control of cutaneous leishmaniasis. *Bulletin de la Société de Pathologie Exotique et de ses Filiales*.

[18] Modabber F (2010). Leishmaniasis vaccines: past, present and future. *International Journal of Antimicrobial Agents*.

[19] Handman E (2001). Leishmaniasis: current status of vaccine development. *Clinical Microbiology Reviews*.

[20] Serebriakov VA, Karakhodzhaeva S, Dzhumaev MD (1972). Effect of leishmanial vaccinations on the dynamics of immunity to diphtheria in conditions of secondary revaccination with adsorbed pertussis-diphtheria-tetanus vaccine. *Meditsinskaya Parazitologiya i Parazitarnye Bolezni*.

[21] Gafurov IM (1999). Experience in controlling and preventing zoonotic cutaneous leishmaniasis in Uzbekistan. *Meditsinskaia Parazitologiia i Parazitarnye Bolezni*.

[22] Tabbara KS, Peters NC, Afrin F (2005). Conditions influencing the efficacy of vaccination with live organisms against *Leishmania major* infection. *Infection and Immunity*.

[23] Noazin S, Modabber F, Khamesipour A (2008). First generation leishmaniasis vaccines: a review of field efficacy trials. *Vaccine*.

[24] Noazin S, Khamesipour A, Moulton LH (2009). Efficacy of killed whole-parasite vaccines in the prevention of leishmaniasis—a meta-analysis. *Vaccine*.

[25] Khalil EAG, Ayed NB, Musa AM (2005). Dichotomy of protective cellular immune responses to human visceral leishmaniasis. *Clinical and Experimental Immunology*.

[26] Musa AM, Khalil EAG, Mahgoub FAE (2008). Immunochemotherapy of persistent post-kala-azar dermal leishmaniasis: a novel approach to treatment. *Transactions of the Royal Society of Tropical Medicine and Hygiene*.

[27] Ghalib H, Modabber F (2007). Consultation meeting on the development of therapeutic vaccines for post kala azar dermal leishmaniasis. *Kinetoplastid Biology and Disease*.

[28] Bhaumik SK, Naskar K, De T (2009). Complete protection against experimental visceral leishmaniasis with complete soluble antigen from attenuated *Leishmania donovani* promastigotes involves Th1-immunity and down-regulation of IL-10. *European Journal of Immunology*.

[29] Bourdoiseau G, Hugnet C, Gonçalves RB (2009). Effective humoral and cellular immunoprotective responses in Li ESAp-MDP vaccinated protected dogs. *Veterinary Immunology and Immunopathology*.

[30] Hommel M, Jaffe CL, Travi B, Milon G (1995). Experimental models for leishmaniasis and for testing anti-leishmanial vaccines. *Annals of Tropical Medicine and Parasitology*.

[31] Requena JM, Soto M, Doria MD, Alonso C (2000). Immune and clinical parameters associated with *Leishmania infantum* infection in the golden hamster model. *Veterinary Immunology and Immunopathology*.

[32] Tesh RB (1995). Control of zoonotic visceral leishmaniasis: is it time to change strategies?. *American Journal of Tropical Medicine and Hygiene*.

[33] Rodríguez-Cortés A, Ojeda A, López-Fuertes L (2007). Vaccination with plasmid DNA encoding KMPII, TRYP, LACK and GP63 does not protect dogs against *Leishmania infantum* experimental challenge. *Vaccine*.

[34] Carrión J, Folgueira C, Alonso C (2008). Immunization strategies against visceral leishmaniosis with the nucleosomal histones of *Leishmania infantum* encoded in DNA vaccine or pulsed in dendritic cells. *Vaccine*.

[35] Dondji B, Pérez-Jimenez E, Goldsmith-Pestana K, Esteban M, McMahon-Pratt D (2005). Heterologous prime-boost vaccination with the LACK antigen protects against murine visceral leishmaniasis. *Infection and Immunity*.

[36] Marques-da-Silva EA, Coelho EAF, Gomes DCO (2005). Intramuscular immunization with p36(LACK) DNA vaccine induces IFN-*γ* production but does not protect BALB/c mice against *Leishmania chagasi* intravenous challenge. *Parasitology Research*.

[37] Gomes DC, Pinto EF, de Melo LD (2007). Intranasal delivery of naked DNA encoding the LACK antigen leads to protective immunity against visceral leishmaniasis in mice. *Vaccine*.

[38] Ramiro MJ, Zárate JJ, Hanke T (2003). Protection in dogs against visceral leishmaniasis caused by *Leishmania infantum* is achieved by immunization with a heterologous prime-boost regime using DNA and vaccinia recombinant vectors expressing LACK. *Vaccine*.

[39] Ramos I, Alonso A, Marcen JM (2008). Heterologous prime-boost vaccination with a non-replicative vaccinia recombinant vector expressing LACK confers protection against canine visceral leishmaniasis with a predominant Th1-specific immune response. *Vaccine*.

[40] Rafati S, Zahedifard F, Nazgouee F (2006). Prime-boost vaccination using cysteine proteinases type I and II of *Leishmania infantum* confers protective immunity in murine visceral leishmaniasis. *Vaccine*.

[41] Rafati S, Nakhaee A, Taheri T (2005). Protective vaccination against experimental canine visceral leishmaniasis using a combination of DNA and protein immunization with cysteine proteinases type I and II of *L. infantum*. *Vaccine*.

[42] Rafati S, Zahedifard F, Azari MK, Taslimi Y, Taheri T (2008). *Leishmania infantum*: prime boost vaccination with C-terminal extension of cysteine proteinase type I displays both type 1 and 2 immune signatures in BALB/c mice. *Experimental Parasitology*.

[43] Khoshgoo N, Zahedifard F, Azizi H, Taslimi Y, Alonso MJ, Rafati S (2008). Cysteine proteinase type III is protective against *Leishmania infantum* infection in BALB/c mice and highly antigenic in visceral leishmaniasis individuals. *Vaccine*.

[44] Wilson ME, Young BM, Andersen KP (1995). A recombinant *Leishmania chagasi* antigen that stimulates cellular immune responses in infected mice. *Infection and Immunity*.

[45] Streit JA, Recker TJ, Donelson JE, Wilson ME (2000). BCG expressing LCR1 of *Leishmania chagasi* induces protective immunity in susceptible mice. *Experimental Parasitology*.

[46] Fragaki K, Suffia I, Ferrua B, Rousseau D, Le Fichoux Y, Kubar J (2001). Immunisation with DNA encoding *Leishmania infantum* protein papLe22 decreases the frequency of parasitemic episodes in infected hamsters. *Vaccine*.

[47] Aguilar-Be I, da Silva Zardo R, Paraguai de Souza E (2005). Cross-protective efficacy of a prophylactic *Leishmania donovani* DNA vaccine against visceral and cutaneous murine leishmaniasis. *Infection and Immunity*.

[48] Oliveira-Freitas E, Casas CP, Borja-Cabrera GP (2006). Acylated and deacylated saponins of Quillaja saponaria mixture as adjuvants for the FML-vaccine against visceral leishmaniasis. *Vaccine*.

[49] Molano I, García Alonso M, Mirón C (2003). A *Leishmania infantum* multi-component antigenic protein mixed with live BCG confers protection to dogs experimentally infected with *L. infantum*. *Veterinary Immunology and Immunopathology*.

[50] Parody N, Soto M, Requena JM, Álonso C (2004). Adjuvant guided polarization of the immune humoral response against a protective multicomponent antigenic protein (Q) from *Leishmania infantum*. A CpG + Q mix protects Balb/c mice from infection. *Parasite Immunology*.

[51] Fernandes AP, Costa MMS, Coelho EAF (2008). Protective immunity against challenge with *Leishmania* (*Leishmania*) *chagasi* in beagle dogs vaccinated with recombinant A2 protein. *Vaccine*.

[52] Zanin FHC, Coelho EAF, Tavares CAP (2007). Evaluation of immune responses and protection induced by A2 and nucleoside hydrolase (NH) DNA vaccines against *Leishmania chagasi* and *Leishmania amazonensis* experimental infections. *Microbes and Infection*.

[53] Lemesre JL, Holzmuller P, Cavaleyra M, Gonçalves RB, Hottin G, Papierok G (2005). Protection against experimental visceral leishmaniasis infection in dogs immunized with purified excreted secreted antigens of *Leishmania infantum* promastigotes. *Vaccine*.

[54] Lemesre J-L, Holzmuller P, Gonçalves RB (2007). Long-lasting protection against canine visceral leishmaniasis using the LiESAp-MDP vaccine in endemic areas of France: double-blind randomised efficacy field trial. *Vaccine*.

[55] Gradoni L, Foglia Manzillo V, Pagano A (2005). Failure of a multi-subunit recombinant leishmanial vaccine (MML) to protect dogs from *Leishmania infantum* infection and to prevent disease progression in infected animals. *Vaccine*.

[56] Coler RN, Goto Y, Bogatzki L, Raman V, Reed SG (2007). Leish-111f, a recombinant polyprotein vaccine that protects against visceral leishmaniasis by elicitation of CD4^+^ T cells. *Infection and Immunity*.

[57] Trigo J, Abbehusen M, Netto EM (2010). Treatment of canine visceral leishmaniasis by the vaccine Leish-111f + MPL-SE. *Vaccine*.

[58] Melby PC, Yang J, Zhao W, Perez LE, Cheng J (2001). *Leishmania donovani* p36(LACK) DNA vaccine is highly immunogenic but not protective against experimental visceral leishmaniasis. *Infection and Immunity*.

[59] Jaffe CL, Rachamim N, Sarfstein R (1990). Characterization of two proteins from *Leishmania donovani* and their use for vaccination against visceral leishmaniasis. *The Journal of Immunology*.

[60] Ghosh A, Zhang WW, Matlashewski G (2001). Immunization with A2 protein results in a mixed Th1/Th2 and a humoral response which protects mice against *Leishmania donovani* infections. *Vaccine*.

[61] Ghosh A, Labrecque S, Matlashewski G (2001). Protection against *Leishmania donovani* infection by DNA vaccination: increased DNA vaccination efficiency through inhibiting the cellular p53 response. *Vaccine*.

[62] Tewary P, Sukumaran B, Saxena S, Madhubala R (2004). Immunostimulatory oligodeoxynucleotides are potent enhancers of protective immunity in mice immunized with recombinant ORFF leishmanial antigen. *Vaccine*.

[63] Tewary P, Jain M, Sahani MH, Saxena S, Madhubala R (2005). A heterologous prime-boost vaccination regimen using ORFF DNA and recombinant ORFF protein confers protective immunity against experimental visceral Leishmaniasis. *Journal of Infectious Diseases*.

[64] Sukumaran B, Tewary P, Saxena S, Madhubala R (2003). Vaccination with DNA encoding ORFF antigen confers protective immunity in mice infected with *Leishmania donovani*. *Vaccine*.

[65] Basu R, Bhaumik S, Basu JM, Naskar K, De T, Roy S (2005). Kinetoplastid membrane protein-11 DNA vaccination induces complete protection against both pentavalent antimonial-sensitive and -resistant strains of *Leishmania donovani* that correlates with inducible nitric oxide synthase activity and IL-4 generation: evidence for mixed Th1- and Th2-like responses in visceral leishmaniasis. *The Journal of Immunology*.

[66] Bhaumik S, Basu R, Sen S, Naskar K, Roy S (2009). KMP-11 DNA immunization significantly protects against L. donovani infection but requires exogenous IL-12 as an adjuvant for comparable protection against L. major. *Vaccine*.

[67] McSorley SJ, Xu D, Liew FY (1997). Vaccine efficacy of Salmonella strains expressing glycoprotein 63 with different promoters. *Infection and Immunity*.

[68] Bhowmick S, Ravindran R, Ali N (2008). Gp63 in stable cationic liposomes confers sustained vaccine immunity to susceptible BALB/c mice infected with *Leishmania donovani*. *Infection and Immunity*.

[69] Bhowmick S, Ravindran R, Ali N (2007). Leishmanial antigens in liposomes promote protective immunity and provide immunotherapy against visceral leishmaniasis via polarized Th1 response. *Vaccine*.

[70] Tewary P, Mehta J, Sukumaran B, Madhubala R (2004). Vaccination with *Leishmania* soluble antigen and immunostimulatory oligodeoxynucleotides induces specific immunity and protection against *Leishmania donovani* infection. *FEMS Immunology and Medical Microbiology*.

[71] Bhowmick S, Ali N (2009). Identification of novel *Leishmania donovani* antigens that help define correlates of vaccine-mediated protection in visceral leishmaniasis. *PLoS One*.

[72] Mazumdar T, Anam K, Ali N (2004). A mixed Th1/Th2 response elicited by a liposomal formulation of *Leishmania* vaccine instructs Th1 responses and resistance to *Leishmania donovani* in susceptible BALB/c mice. *Vaccine*.

[73] Bhardwaj S, Vasishta RK, Arora SK (2009). Vaccination with a novel recombinant *Leishmania* antigen plus MPL provides partial protection against L. donovani challenge in experimental model of visceral leishmaniasis. *Experimental Parasitology*.

[74] Carter KC, Henriquez FL, Campbell SA (2007). DNA vaccination against the parasite enzyme gamma-glutamylcysteine synthetase confers protection against *Leishmania donovani* infection. *Vaccine*.

[75] Palatnik-de-Sousa CB, Paraguai-de-Souza E, Gomes EM, Borojevic R (1994). Experimental murine *Leishmania donovani* infection: immunoprotection by the fucose-mannose ligand (FML). *Brazilian Journal of Medical and Biological Research*.

[76] Santos WR, Paraguai de Souza E, Palatnik M, Palatnik de Sousa CB (1999). Vaccination of Swiss Albino mice against experimental visceral leishmaniasis with the FML antigen of *Leishmania donovani*. *Vaccine*.

[77] Borja-Cabrera GP, Correia Pontes NN, da Silva VO (2002). Long lasting protection against canine kala-azar using the FML-QuilA saponin vaccine in an endemic area of Brazil (São Gonçalo do Amarante, RN). *Vaccine*.

[78] Saraiva EM, de Figueiredo Barbosa A, Santos FN (2006). The FML-vaccine (Leishmune) against canine visceral leishmaniasis: a transmission blocking vaccine. *Vaccine*.

[79] Nogueira FS, Moreira MAB, Borja-Cabrera GP (2005). Leishmune vaccine blocks the transmission of canine visceral leishmaniasis: absence of *Leishmania* parasites in blood, skin and lymph nodes of vaccinated exposed dogs. *Vaccine*.

[80] Saldarriaga OA, Travi BL, Park W, Perez LE, Melby PC (2006). Immunogenicity of a multicomponent DNA vaccine against visceral leishmaniasis in dogs. *Vaccine*.

[81] Tonui WK, Mpoke SS, Orago AS, Turco SJ, Mbati PA, Mkoji GM (2003). *Leishmania donovani*-derived lipophosphoglycan plus BCG induces a Th1 type immune response but does not protect Syrian golden hamsters (*Mesocricetus auratus*) and BALB/c mice against *Leishmania donovani*. *Onderstepoort Journal of Veterinary Research*.

[82] Rachamim N, Jaffe CL (1993). Pure protein from *Leishmania donovani* protects mice against both cutaneous and visceral leishmaniasis. *The Journal of Immunology*.

[83] Coler RN, Reed SG (2005). Second-generation vaccines against leishmaniasis. *Trends in Parasitology*.

[84] Bertholet S, Goto Y, Carter L (2009). Optimized subunit vaccine protects against experimental leishmaniasis. *Vaccine*.

[85] Chakravarty J, Kumar S, Trivedi S (2011). A clinical trial to evaluate the safety and immunogenicity of the LEISH-F1+MPL-SE vaccine for use in the prevention of visceral leishmaniasis. *Vaccine*.

[86] Gurunathan S, Klinman DM, Seder RA (2000). DNA vaccines: immunology, application, and optimization. *Annual Review of Immunology*.

[87] Rodrigues MM, Boscardin SB, Vasconcelos JR, Hiyane MI, Salay G, Soares IS (2003). Importance of CD8 T cell-mediated immune response during intracellular parasitic infections and its implications for the development of effective vaccines. *Anais da Academia Brasileira de Ciencias*.

[88] Suffia I, Ferrua B, Stien X (2000). A novel *Leishmania infantum* recombinant antigen which elicits interleukin 10 production by peripheral blood mononuclear cells of patients with visceral leishmaniasis. *Infection and Immunity*.

[89] Nylén S, Sacks D (2007). Interleukin-10 and the pathogenesis of human visceral leishmaniasis. *Trends in Immunology*.

[90] Gurunathan S, Sacks DL, Brown DR (1997). Vaccination with DNA encoding the immunodominant LACK parasite antigen confers protective immunity to mice infected with *Leishmania major*. *Journal of Experimental Medicine*.

[91] Gurunathan S, Prussin C, Sacks DL, Seder RA (1998). Vaccine requirements for sustained cellular immunity to an intracellular parasitic infection. *Nature Medicine*.

[92] Gurunathan S, Stobie L, Prussin C (2000). Requirements for the maintenance of Th1 immunity in vivo following DNA vaccination: a potential immunoregulatory role for CD8^+^ T cells. *The Journal of Immunology*.

[93] Gonzalo RM, Del Real G, Rodriguez JR (2002). A heterologous prime-boost regime using DNA and recombinant vaccinia virus expressing the *Leishmania infantum* P36/LACK antigen protects BALB/c mice from cutaneous leishmaniasis. *Vaccine*.

[94] Rafati S, Fasel N, Masina S (2003). Leishmania cysteine proteinases: from gene to subunit vaccine. *Current Genomics*.

[95] Mazumder S, Maji M, Das A, Ali N (2011). Potency, efficacy and durability of DNA/DNA, DNA/ protein and protein/protein based vaccination using gp63 against *Leishmania donovani* in BALB/c mice. *PLoS One*.

[96] Sinha S, Sundaram S, Singh AP, Tripathi A (2001). A gp63 based vaccine candidate against Visceral Leishmaniasis. *Bioinformation*.

[97] Moll H, Berberich C (2001). Dendritic cell-based vaccination strategies: induction of protective immunity against leishmaniasis. *Immunobiology*.

[98] Brandonisio O, Spinelli R, Pepe M (2004). Dendritic cells in *Leishmania* infection. *Microbes and Infection*.

[99] Vanloubbeeck YF, Ramer AE, Jie F, Jones DE (2004). CD4^+^ Th1 cells induced by dendritic cell-based immunotherapy in mice chronically infected with *Leishmania amazonensis* do not promote healing. *Infection and Immunity*.

[100] Ahuja SS, Reddick RL, Sato N (1999). Dendritic cell (DC)-based anti-infective strategies: DCs engineered to secrete IL-12 are a potent vaccine in a murine model of an intracellular infection. *The Journal of Immunology*.

[101] Ghosh M, Pal C, Ray M, Maitra S, Mandal L, Bandyopadhyay S (2003). Dendritic cell-based immunotherapy combined with antimony-based chemotherapy cures established murine visceral leishmaniasis. *The Journal of Immunology*.

[102] Moore EM, Lockwood DN (2010). Treatment of visceral leishmaniasis. *Journal of Global Infectious Diseases*.

[103] Bhowmick S, Mazumdar T, Sinha R, Ali N (2010). Comparison of liposome based antigen delivery systems for protection against *Leishmania donovani*. *Journal of Controlled Release*.

[104] Henriquez FL, Campbell SA, Roberts CW, Mullen AB, Burchmore R, Carter KC (2010). Vaccination with recombinant *Leishmania donovani* gamma-glutamylcysteine synthetase fusion protein protects against L. donovani infection. *Journal of Parasitology*.

[105] Peters NC, Kimblin N, Secundino N, Kamhawi S, Lawyer P, Sacks DL (2009). Vector transmission of *Leishmania* abrogates vaccine-induced protective immunity. *PLoS Pathogens*.

[106] Gomes RB, Brodskyn C, de Oliveira CI (2002). Seroconversion against Lutzomyia longipalpis saliva concurrent with the development of anti-*Leishmania chagasi* delayed-type hypersensitivity. *Journal of Infectious Diseases*.

[107] Fishbein DB, Robinson LE (1993). Rabies. *The New England Journal of Medicine*.

[108] Sauret JM, Vilissova N (2002). Human brucellosis. *Journal of the American Board of Family Practice*.

[109] Postigo JAR (2010). Leishmaniasis in the World Health Organization Eastern Mediterranean Region. *International Journal of Antimicrobial Agents*.

[110] Ashford RW (2000). The leishmaniases as emerging and reemerging zoonoses. *International Journal for Parasitology*.

[111] Biglino A, Bolla C, Concialdi E, Trisciuoglio A, Romano A, Ferroglio E (2010). Asymptomatic *Leishmania infantum* infection in an area of northwestern Italy (Piedmont region) where such infections are traditionally nonendemic. *Journal of Clinical Microbiology*.

[112] Schantz PM, Steurer FJ, Duprey ZH (2005). Autochthonous visceral leishmaniasis in dogs in North America. *Journal of the American Veterinary Medical Association*.

[113] Ready PD (2010). Leishmaniasis emergence in Europe. *Euro Surveillance*.

[114] Baneth G, Shaw SE (2002). Chemotherapy of canine leishmaniosis. *Veterinary Parasitology*.

[115] Palatnik-de-Sousa CB, dos Santos WR, França-Silva JC (2001). Impact of canine control on the epidemiology of canine and human visceral leishmaniasis in Brazil. *American Journal of Tropical Medicine and Hygiene*.

[116] Reithinger R, Coleman PG, Alexander B, Vieira EP, Assis G, Davies CR (2004). Are insecticide-impregnated dog collars a feasible alternative to dog culling as a strategy for controlling canine visceral leishmaniasis in Brazil?. *International Journal for Parasitology*.

[117] Courtenay O, Quinnell RJ, Garcez LM, Shaw JJ, Dye C (2002). Infectiousness in a cohort of Brazilian dogs: why culling fails to control visceral leishmaniasis in areas of high transmission. *Journal of Infectious Diseases*.

[118] Boggiatto PM, Ramer-Tait AE, Metz K (2010). Immunologic indicators of clinical progression during canine *Leishmania infantum* infection. *Clinical and Vaccine Immunology*.

[119] Poot J, Janssen LHM, van Kasteren-Westerneng TJ, van der Heijden-Liefkens KHA, Schijns VEJC, Heckeroth A (2009). Vaccination of dogs with six different candidate leishmaniasis vaccines composed of a chimerical recombinant protein containing ribosomal and histone protein epitopes in combination with different adjuvants. *Vaccine*.

[120] Bahia D, Gontijo NF, León IR (2007). Antibodies from dogs with canine visceral leishmaniasis recognise two proteins from the saliva of Lutzomyia longipalpis. *Parasitology Research*.

[121] Giunchetti RC, Corrêa-Oliveira R, Martins-Filho OA (2008). A killed* Leishmania* vaccine with sand fly saliva extract and saponin adjuvant displays immunogenicity in dogs. *Vaccine*.

[122] Gomes R, Teixeira C, Teixeira MJ (2008). Immunity to a salivary protein of a sand fly vector protects against the fatal outcome of visceral leishmaniasis in a hamster model. *Proceedings of the National Academy of Sciences of the United States of America*.

[123] Collin N, Gomes R, Teixeira C (2009). Sand fly salivary proteins induce strong cellular immunity in a natural reservoir of visceral leishmaniasis with adverse consequences for *Leishmania*. *PLoS Pathogens*.

[124] Dantas-Torres F (2006). Leishmune vaccine: the newest tool for prevention and control of canine visceral leishmaniosis and its potential as a transmission-blocking vaccine. *Veterinary Parasitology*.

[125] Palatnik-de-Sousa CB, Dutra HS, Borojevic R (1993). *Leishmania donovani* surface glycoconjugate GP36 is the major immunogen component of the Fucose-Mannose Ligand (FML). *Acta Tropica*.

[126] da Silva VO, Borja-Cabrera GP, Correia Pontes NN (2000). A phase III trial of efficacy of the FML-vaccine against canine kala-azar in an endemic area of Brazil (São Gonçalo do Amaranto, RN). *Vaccine*.

[127] Borja-Cabrera GP, Mendes AC, Paraguai de Souza E (2004). Effective immunotherapy against canine visceral leishmaniasis with the FML-vaccine. *Vaccine*.

[128] Borja-Cabrera GP, Santos FN, Santos FB (2010). Immunotherapy with the saponin enriched-Leishmune vaccine versus immunochemotherapy in dogs with natural canine visceral leishmaniasis. *Vaccine*.

[129] Palatnik-de-Sousa CB, Silva-Antunes I, Morgado Ade A, Menz I, Palatnik M, Lavor C (2009). Decrease of the incidence of human and canine visceral leishmaniasis after dog vaccination with Leishmune in Brazilian endemic areas. *Vaccine*.

[130] Resende DM, Caetano BC, Dutra MS (2008). Epitope mapping and protective immunity elicited by adenovirus expressing the *Leishmania* amastigote specific A2 antigen: correlation with IFN-*γ* and cytolytic activity by CD8^+^ T cells. *Vaccine*.

[131] Daneshvar H, Molaei MM, Kamiabi H, Burchmore R, Hagan P, Phillips RS (2010). Gentamicin-attenuated *Leishmania infantum*: cellular immunity production and protection of dogs against experimental canine leishmaniasis. *Parasite Immunology*.

[132] Mitchell GF, Handman E, Spithill TW (1984). Vaccination against cutaneous Leishmaniasis in mice using nonpathogenic cloned promastigotes of *Leishmania major* and importance of route of injection. *Australian Journal of Experimental Biology and Medical Science*.

[133] Daneshvar H, Coombs GH, Hagan P, Phillips RS (2003). *Leishmania mexicana* and *Leishmania major*: attenuation of wild-type parasites and vaccination with the attenuated lines. *Journal of Infectious Diseases*.

[134] Aebischer T, Morris L, Handman E (1994). Intravenous injection of irradiated *Leishmania major* into susceptible BALB/c mice: immunization or protective tolerance. *International Immunology*.

[135] Gorczynski RM (1985). Immunization of susceptible BALB/c mice against Leishmania braziliensis. II. Use of temperature-sensitive avirulent clones of parasite for vaccination purposes. *Cellular Immunology*.

[136] Beverley SM (2003). Protozomics: trypanosomatid parasite genetics comes of age. *Nature Reviews Genetics*.

[137] Titus RG, Gueiros-Filho FJ, de Freitas LAR, Beverley SM (1995). Development of a safe live *Leishmania* vaccine line by gene replacement. *Proceedings of the National Academy of Sciences of the United States of America*.

[138] Amaral VF, Teva A, Oliveira-Neto MP (2002). Study of the safety, immunogenicity and efficacy of attenuated and killed *Leishmania* (*Leishmania*) major vaccines in a rhesus monkey (Macaca mulatta) model of the human disease. *Memorias do Instituto Oswaldo Cruz*.

[139] Muyombwe A, Olivier M, Ouellette M, Papadopoulou B (1997). Selective killing of *Leishmania* amastigotes expressing a thymidine kinase suicide gene. *Experimental Parasitology*.

[140] Davoudi N, Tate CA, Warburton C, Murray A, Mahboudi F, McMaster WR (2005). Development of a recombinant *Leishmania major* strain sensitive to ganciclovir and 5-fluorocytosine for use as a live vaccine challenge in clinical trials. *Vaccine*.

[141] Sah JF, Ito H, Kolli BK, Peterson DA, Sassa S, Chang KP (2002). Genetic rescue of *Leishmania* deficiency in porphyrin biosynthesis creates mutants suitable for analysis of cellular events in uroporphyria and for photodynamic therapy. *Journal of Biological Chemistry*.

[142] Kumari S, Samani M, Khare P (2009). Photodynamic vaccination of hamsters with inducible suicidal mutants of *Leishmania amazonensis* elicits immunity against visceral leishmaniasis. *European Journal of Immunology*.

[143] Ouellette M, Drummelsmith J, El Fadili A, Kündig C, Richard D, Roy G (2002). Pterin transport and metabolism in *Leishmania* and related trypanosomatid parasites. *International Journal for Parasitology*.

[144] Papadopoulou B, Roy G, Breton M (2002). Reduced infectivity of a *Leishmania donovani* biopterin transporter genetic mutant and its use as an attenuated strain for vaccination. *Infection and Immunity*.

[145] Selvapandiyan A, Duncan R, Debrabant A (2006). Genetically modified live attenuated parasites as vaccines for leishmaniasis. *Indian Journal of Medical Research*.

[146] Selvapandiyan A, Dey R, Nylen S, Duncan R, Sacks D, Nakhasi HL (2009). Intracellular replication-deficient *Leishmania donovani* induces long lasting protective immunity against visceral leishmaniasis. *The Journal of Immunology*.

[147] Silvestre R, Cordeiro-da-Silva A, Santarém N, Vergnes B, Sereno D, Ouaissi A (2007). SIR2-deficient *Leishmania infantum* induces a defined IFN-*γ*/IL-10 pattern that correlates with protection. *The Journal of Immunology*.

[148] Taylor VM, Muñoz DL, Cedeño DL, Vélez ID, Jones MA, Robledo SM (2010). *Leishmania tarentolae*: utility as an in vitro model for screening of antileishmanial agents. *Experimental Parasitology*.

[149] Breton M, Tremblay MJ, Ouellette M, Papadopoulou B (2005). Live nonpathogenic parasitic vector as a candidate vaccine against visceral leishmaniasis. *Infection and Immunity*.

[150] Azizi H, Hassani K, Taslimi Y, Najafabadi HS, Papadopoulou B, Rafati S (2009). Searching for virulence factors in the non-pathogenic parasite to humans *Leishmania tarentolae*. *Parasitology*.

[151] Mizbani A, Taheri T, Zahedifard F (2009). Recombinant *Leishmania tarentolae* expressing the A2 virulence gene as a novel candidate vaccine against visceral leishmaniasis. *Vaccine*.

[152] Beattie L, Evans KJ, Kaye PM, Smith DF (2008). Transgenic *Leishmania* and the immune response to infection. *Parasite Immunology*.

[153] Rapsodi against leishmaniasis. http://www.fp7-rapsodi.eu/.

[154] Development of a DNA vaccine for visceral leishmaniasis: LEISHDNAVAX. http://www.leishdnavax.org/.

